# Structural Variants and Selective Sweep Foci Contribute to Insecticide Resistance in the *Drosophila* Genetic Reference Panel

**DOI:** 10.1534/g3.118.200619

**Published:** 2018-09-04

**Authors:** Paul Battlay, Pontus B. Leblanc, Llewellyn Green, Nandita R. Garud, Joshua M. Schmidt, Alexandre Fournier-Level, Charles Robin

**Affiliations:** * School of BioSciences, The University of Melbourne, Parkville, 3010, Victoria, Australia; † Gladstone Institute, University of California, San Francisco, CA, 94158; ‡ Department of Evolutionary Genetics, Max Planck Institute for Evolutionary Anthropology, Leipzig, 04103, Saxony, Germany

**Keywords:** Drosophila Genetic Reference Panel (DGRP), malathion, permethrin, acetylcholinesterase, Cyp6a17

## Abstract

Patterns of nucleotide polymorphism within populations of *Drosophila melanogaster* suggest that insecticides have been the selective agents driving the strongest recent bouts of positive selection. However, there is a need to explicitly link selective sweeps to the particular insecticide phenotypes that could plausibly account for the drastic selective responses that are observed in these non-target insects. Here, we screen the Drosophila Genetic Reference Panel with two common insecticides; malathion (an organophosphate) and permethrin (a pyrethroid). Genome-wide association studies map survival on malathion to two of the largest sweeps in the *D. melanogaster* genome; *Ace* and *Cyp6g1*. Malathion survivorship also correlates with lines which have high levels of *Cyp12d1*, *Jheh1* and *Jheh2* transcript abundance. Permethrin phenotypes map to the largest cluster of P450 genes in the Drosophila genome, however in contrast to a selective sweep driven by insecticide use, the derived allele seems to be associated with susceptibility. These results underscore previous findings that highlight the importance of structural variation to insecticide phenotypes: *Cyp6g1* exhibits copy number variation and transposable element insertions, *Cyp12d1* is tandemly duplicated, the *Jheh* loci are associated with a *Bari1* transposable element insertion, and a *Cyp6a17* deletion is associated with susceptibility.

Understanding the genetic basis of insecticide resistance is important, not only to inform the implementation of insecticides in agriculture and disease vector control, but also as an evolutionary case-study operating over observable periods of time. Utilizing genome-wide association studies (GWAS) to investigate insecticide resistance provides an unbiased way to identify multiple natural genetic variants associated with a phenotype, while the polymorphism data surrounding associated variants may provide clues to their evolutionary trajectory.

Since its introduction in 2012, the Drosophila Genetic Reference Panel (DGRP; Mackay *et al.* 2012) has proved to be a powerful tool for dissecting the genetic architecture of a range of *Drosophila melanogaster* phenotypes through the implementation of genome-wide association studies (GWAS) on the DGRP’s 205 inbred, sequenced lines. Insecticide-induced mortality has been among these phenotypes (Battlay *et al.* 2016; Denecke *et al.* 2017; Schmidt *et al.* 2017). In 2015, the DGRP’s utility was increased with the introduction of transcriptome data (Huang *et al.* 2015), allowing phenotypes to be tested for association directly with variation in individual transcripts across the *D. melanogaster* transcriptome.

The sequence data generated by the DGRP has also proved to be a valuable resource for the study of population genomics, and has allowed the identification of regions of strong, recent selection in the DGRP’s ancestral population (Garud *et al.* 2015). Two of the most pronounced of these signals, genome wide, come from insecticide resistance loci *Cyp6g1* and *Ace*. Significant selective signals have also been identified around these loci in other *D. melanogaster* populations (Garud and Petrov 2016), and related species (Signor *et al.* 2017; *D. simulans*), as well as by targeted analyses in *D. melanogaster* of selection at *Ace* (Karasov, Messer and Petrov 2010) and *Cyp6g1* (Catania *et al.* 2004; Schmidt *et al.* 2010).

The fact that insecticides appear to have played such an important role in the recent evolutionary history of the DGRP allows us the rare opportunity to study the quantitative genetics of a trait in the process of strong selection. It is unknown, however, what compound or compounds are causing this selection. Although natural variation in both *Ace* and *Cyp6g1* has been demonstrated to confer resistance to various insecticides, attempts to detect these associations in the DGRP, and hence associate the selective sweeps at these loci with a particular compound, have departed from expectations.

Acetylcholineesterase (Ace) is the molecular target of organophosphate insecticides, and four non-synonymous substitutions in the enzyme’s active-site groove have been demonstrated to reduce the binding capacity of organophosphate insecticides (Menozzi *et al.* 2004). Battlay *et al.* (2016) were, however, unable to detect a significant effect of variation in *Ace* on resistance to the organophosphate azinphos-methyl in DGRP larvae, but instead detected a strong association with alleles that overexpressed *Cyp6g1*, a cytochrome P450 enzyme previously shown to confer metabolic resistance to DDT and imidacloprid when overexpressed (Daborn *et al.* 2001, Daborn *et al.* 2002, Joußen *et al.* 2008, Hoi *et al.* 2014). Although the link between natural alleles which overexpress *Cyp6g1* and resistance to DDT has been demonstrated in a worldwide sample (Catania *et al.* 2004) and Australian populations (Schmidt *et al.* 2010), a similar result was not observed in the DGRP (Schmidt *et al.* 2017).

Aside from the recently reported association between azinphos-methyl resistance and *Cyp6g1* in the DGRP (Battlay *et al.* 2016), previous investigations have mapped organophosphate resistance to a region including *Cyp6g1* (Kikkawa 1961 [parathion]; Pyke *et al.* 2004 [diazinon]). Cross-resistance to the organophosphate malathion was reported at the mapping region on chromosome 2 by Kikkawa (1961), and Ogita’s (1958) mapping of DDT resistance. Le Goff *et al.* (2003) also reported malathion cross-resistance in DDT resistant lab lines (*Hikone-R* and *Wisconsin*), both of which showed heightened levels of *Cyp6g1* transcript. Likewise, DDT-resistant *91-R* (which carries a resistance allele at the *Cyp6g1* locus [Schmidt *et al.* 2017] and overexpresses the enzyme [Pedra *et al.* 2004]), shows cross resistance to malathion (Misra *et al.* 2013).

In light of this evidence, resistance to organophosphates makes a compelling subject for study in the DGRP. Natural variants with the two strongest signals of selection in the population, *Ace* and *Cyp6g1*, may both confer resistance to these compounds, and the organophosphate class of insecticides has been employed widely over a long period of time, giving it opportunity to induce such selective pressures.

Pyrethroids have also been extensively utilized both spatially and temporally in insect control, however, natural variation contributing to pyrethroid insecticide class resistance in *D. melanogaster* is less well understood. Like Ace, resistance-causing mutations in the molecular target of pyrethroids and DDT, the voltage-gated sodium channel, are common in insect pest species (Dong *et al.* 2014). However, orthologous mutations have not been described as natural variants in *D. melanogaster*, although EMS mutagenesis has yielded mutations in *para* (the *D. melanogaster* voltage-gated sodium channel alpha subunit) that cause resistance to DDT and the pyrethroid deltamethrin (Pittendrigh *et al.* 1997). At least one *D. melanogaster* cytochrome P450 gene has been shown to be involved in pyrethroid biology. *Cyp4e3* is both induced in response to permethrin exposure, and capable of increasing resistance to the insecticide when overexpressed (Terhzaz *et al.* 2015), however, once again natural variation in this gene has not been described, and any contribution to of this locus to pyrethroid resistance in wild populations is yet to be determined.

Organophosphates and pyrethroids are two of the oldest and most widely used insecticide classes in the world today. Here, we investigate the genetic basis of resistance in the DGRP to a representative of each of these classes; the organophosphate, malathion, and the pyrethroid, permethrin. We assess genomic and transcriptomic associations with both male and female adults at multiple doses and incorporate genotyping of structural variation and previously identified signatures of selective sweeps.

## Materials and Methods

### Fly lines

DGRP lines were generated by Mackay *et al.* (2012) and obtained from the Bloomington Drosophila stock center in Indiana. The *6g1*HR-GAL4 driver line was generated by Chung *et al.* (2007). The RAL_517 *Cyp6g1*-KO line was generated by Denecke *et al.* (2017). The UAS-*Cyp12d1* line was generated by Daborn *et al.* (2007). *Cyp6a17^KG04448^* flies were generated by Bellen *et al.* (2011) and were obtained from the Bloomington Drosophila Stock Center. All fly stocks were maintained at 25° on rich medium containing, maltose (46g/L), dextrose (75g/L), yeast (35g/L), soy flour (20g/L), maize meal (73g/L), agar (6g/L), acid mix (14ml/L), and tegosept (16ml/L). The acid mix solution was made up of orthophosphoric acid (42ml/L), and propionic acid (412ml/L), while the tegosept solution was 50g tegosept dissolved in 950 ml of 95% EtOH.

### Insect bioassays

Adult flies for bioassays were anesthetized with CO_2_ at 0-24 hr after eclosion and sorted by sex into holding vials containing rich media, where they were kept for 3-4 days, resulting in 3-5 day old adults for use in bioassays. Assays commenced between 11 am and 12 pm. 20mL glass scintillation vials were treated with 500μl of acetone/insecticide solution at the required concentration and rolled using a hotdog warmer (heat off) until the acetone had evaporated. ∼7 flies were transferred to each vial without the use of anesthesia, and cotton wool moistened with 10% sucrose solution was used to stopper the scintillation vials. DGRP lines were screened at a single dose.

### Phenotype selection

Twenty-four-hour mortality in adult insects was selected as a phenotype as this is a standard assay in *D. melanogaster* insecticide resistance literature (Daborn *et al.* 2001; Schmidt *et al.* 2010; Schmidt *et al.* 2017), and DGRP lines show negligible control mortality in scintillation vial bioassays at this time point (Schmidt *et al.* 2017). ‘Knockdown”, a phenotype in which the fly lies paralyzed and twitching, or exhibits uncontrolled flight, is a field-relevant effect of pyrethroid insecticides. We therefore also measured incidence of this phenotype at three hours in permethrin-treated flies. As no pronounced knockdown effect was expected (or observed) in malathion-treated flies, we instead scored mortality at additional time points (3, 6 and 12 hr). Discriminant insecticide doses (1μg/vial for malathion, 10μg/vial for permethrin) were identified by screening 20 randomly selected DGRP lines on a range of concentrations. Transgenic lines were screened at multiple doses and scored for mortality at 24 hr. A minimum of three biological replicates were performed for each sex of each DGRP line, and for each sex at each dose for transgenic lines.

### Calculation of LD_50_

For transgenic lines, linear models were fitted to dose-mortality data on a log-probit scale using ‘glm’ in R (R Core Team 2013) and scripts from Johnson *et al.* (2013). Median lethal dose (LD_50_) values and 95% confidence intervals were calculated using Fieller’s method from fitted linear models (Finney 1971).

### Estimation of broad-sense heritability and sex effect

The broad-sense heritability of each phenotype was estimated for both sexes individually as *σ^2^_G_ / (σ^2^_G_ + σ^2^_E_)*, using the variance components of a linear model of the form *phenotype ∼1 + (1 | line)* using ‘lme4’ in R (R Core Team 2013). Male sex effect was estimated from the male sex intercept of the model *phenotype ∼ sex + (0 + sex | line)* using ‘lme4’ in R (R Core Team 2013).

### Genome-wide association studies

Phenotype files for 170 DGRP lines, consisting of mean mortality data for both males and females, were generated for all phenotypes and were submitted to the Mackay lab DGRP2 GWAS pipeline (http://dgrp2.gnets.ncsu.edu/; Huang *et al.* 2014). The genome wide significance threshold (1×10^−5^) was corrected for the number of phenotypes tested for each insecticide (8 and 4 for malathion and permethrin respectively) and applied to the Mackay lab pipeline ‘mixed p-value’ (association after correction for the effects of wolbachia and major chromosomal inversions). Bonferroni significance thresholds were calculated as 0.05 divided by the product of the number of genomic variants (1,877,810 and 1,876,330 for malathion and permethrin respectively) and phenotypes tested.

### Assessment of variants within H12 peaks

Malathion associated variants were identified in four H12 selective sweep peaks. To test whether these variants may be the foci of these sweeps, distortions in candidate allele frequency in lines sharing the two most common haplotypes (H1 and H2) at each H12 peak were tested using Fisher’s exact test.

### Transcriptome to phenotype associations

Transcriptome data for 1-3 day old adult flies from 185 DGRP lines were recovered from the DGRP website (http://dgrp2.gnets.ncsu.edu/data.html; Huang *et al.* 2015). Mean transcription level was calculated for each gene in each sex from two biological replicates, to give a mean level for each of the 18,139 transcripts measured by Huang *et al.* (2015) in each DGRP line, for both males and females. A linear model was fit between mean transcription level of each gene measured by Huang *et al.* (2015) for the relevant sex, and each malathion and permethrin phenotype individually. 1×10^−3^ (which roughly corresponds to the genome-wide significance threshold used in GWAS adjusted for the smaller number of tests performed against the transcriptome compared to the genome) was used as a base significance threshold for transcriptome associations after correction for the number of phenotypes tested for each insecticide (8 and 4 for malathion and permethrin respectively). Bonferroni significance thresholds were calculated as 0.05 divided by the product of the number of transcripts (18139) and phenotypes tested. Associated variants from GWAS were also tested for annotation as eQTL using data from Huang *et al.* 2015.

### Genotyping of structural variation

BAM files containing alignments of DGRP line sequences from Illumina platforms to the *y*; *cn bw sp*; reference genome were recovered from the Baylor College of Medicine website (https://www.hgsc.bcm.edu/content/dgrp-lines; Mackay *et al.* 2012). Local alignments at candidate loci were visualized with IGV 2.0 software (Robinson *et al.* 2011) to manually score structural variation. *Cyp6g1*, *Cyp6a17/23*, and *Cyp12d1* structural variants were previously genotyped in Good *et al.* (2014).

Genotyping of the *Bari1* insertion presence upstream of *Jheh1* and *Jheh2* genes was provided by Josefa González derived from diagnostic PCR (33 lines; González, Macpherson and Petrov 2009) and T-lex software (119 lines; Fiston-Lavier *et al.* 2010). These datasets were supplemented with our own manual calling of the insertion using IGV 2.0 software (167 lines; Thorvaldsdóttir *et al.* 2013). This resulted in 80 DGRP lines with high confidence (at least two concurrent calls) *Bari-Jheh* genotypes that also had matching transcriptome and malathion phenotype data, which were used for further analysis.

Amplification events involving *Cyp6g1* and *Cyp6g2* were inferred from local read depth in DGRP BAM files. Read depth at each nucleotide position under consideration were recovered using the Genome Analysis Toolkit ‘DepthOfCoverage’ utility (McKenna *et al.* 2010). Regions interrogated were non-overlapping portions of the *Cyp6g1* amplicon (2R:8072727-8074976), the *Cyp6g1g2* amplicon (2R:8075688-8077656), and a control region of similar size just upstream of *Cyp6g1* which does not exhibit structural variation in the DGRP (2R:8070657-8072656). Mean read depth for each amplicon was calculated for each DGRP line, and normalized to mean read depth of the control region.

### Transgenic overexpression

*Cyp12d1* was overexpressed using the GAL4/UAS system (Brand and Perrimon 1993) and the *6g1*HR-GAL4 driver described by Chung *et al.* (2007). *6g1*HR-GAL4 virgin females, in which GAL4 is regulated by *Cyp6g1* upstream sequence originating from Hikone-R line flies, were crossed to males carrying an additional copy of *Cyp12d1* under control of a UAS promoter. w^1118^ was used as a control (Daborn *et al.* 2007).

### Frequencies of Ace and Cyp6g1 in the Drosophila Genome Nexus

FASTA files from the Drosophila Genome Nexus release 1.1 (Lack *et al.* 2016) were downloaded from http://www.johnpool.net/genomes.html. The provided scripts were used to mask data for identity by descent and population admixture. Variants were retrieved from the genomes using the provided dataslice.pl script. In the case of *Cyp6g1*, we used 2R:8072837, a SNP in complete linkage disequilibrium with derived alleles of *Cyp6g1* in the DGRP, as a marker for derived *Cyp6g1* alleles in the DGN data.

### Data Availability

The DGRP strains are available from Bloomington Stock center. All data are reported in the manuscript or in the associated supplementary material. DGRP phenotypes and raw Mackay pipeline outputs for each phenotype are supplied in the supplementary data file. Supplemental material available at Figshare: https://doi.org/10.25387/g3.6954443.

## Results

### Phenotypes

Male and female insecticide phenotypes (3, 6, 12 and 24-hour malathion mortality at 1μg/vial, permethrin 3-hour knockdown and permethrin 24-hour mortality at 10 μg/vial) were measured for 170 DGRP lines. Malathion broad-sense heritability (H^2^) ranged from 0.56-0.68, while permethrin H^2^ ranged from 0.56-0.61 (Table S1). For all insecticide phenotypes, males showed higher population mean susceptibility than females, and the effect of sex on phenotype was greater for permethrin (0.11-0.24) than malathion (0.02-0.04; Table S1).

### Genome-wide association studies

Phenotypes were tested for associations with genomic variants using the DGRP2 pipeline, which corrects for the effects on phenotype of wolbachia infection status and five common chromosomal inversion genotypes in each DGRP line (Huang *et al.* 2014). Wolbachia infection significantly reduced insecticide susceptibility to both insecticides at multiple phenotypes (*P* < 0.05; Table S1). P-values arising from these mixed linear models are reported as ‘mixed p-values’, and two thresholds were utilized in the assessment of significant associations: The ‘genome-wide significance threshold’ (1×10^−5^), corrected for the number of phenotypes tested in each insecticide, and the Bonferroni significance threshold (0.05 corrected for the number of DGRP variants and phenotypes tested).

**Figure 1:**
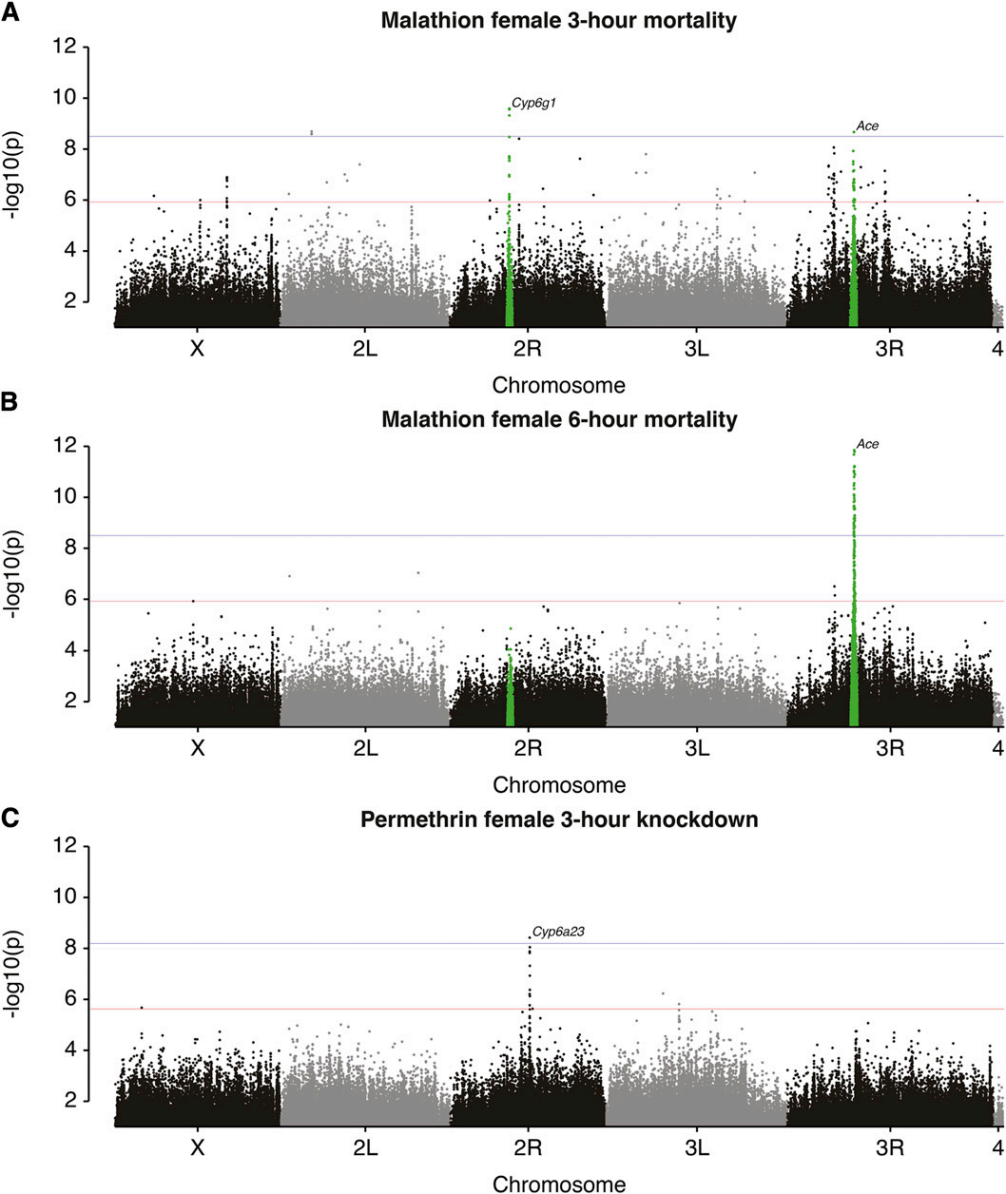
Most significant DGRP genomic variant associations with malathion and permethrin phenotypes. Manhattan plots (mixed p-value against genomic location) for two malathion phenotypes and one permethrin phenotype, showing strong associations around *Cyp6g1*, *Ace*, and members of a cluster of cytochrome P450s on chromosome 2R. Genome-wide significance thresholds are indicated in red, Bonferroni significance thresholds are indicated in blue. Green highlights on malathion Manhattan plots show variants within H12 selective sweep statistic peaks identified around *Cyp6g1* and *Ace* (Garud *et al.* 2015).

Across malathion phenotypes, 273 unique variants were identified with mixed p-values below the genome-wide significance threshold (1.25×10^−6^; Figure 1A, Figure 1B, Figure S1A, Table S2, Table S4), more than half of which (176) were only associated with a single sex. 12 nonsynonymous variants in 8 genes were associated with malathion phenotypes, including three in *Ace*, the molecular target of organophosphate insecticides. 62 variants were associated with mixed p-values below the Bonferroni-corrected significance threshold (3.33×10^−9^). Enrichment analyses using gene function, protein interactions and pathway relations failed to identify significant terms with the malathion phenotypes (Antonov 2011; Table S2).

For permethrin, 39 variants were associated with any phenotype with mixed p-values below the genome wide significance threshold (2.5×10^−6^; Figure 1C, Figure S1B, Table S5, Table S6), two of which achieved Bonferroni-corrected significance (*P* < 6.66×10^−9^). Eight of the nine variants common to both sexes, including the Bonferroni-significant associations, were identified in the cytochrome P450 cluster on the right arm of chromosome 2, and consequently P450 related terms were significant in gene ontology enrichment analyses (Antonov 2011; Table S2).

### Selective sweeps at resistance loci

Variants that are indicative of previously described resistance haplotypes surrounding *Ace* and *Cyp6g1* were among those strongly associated with malathion phenotypes (Figure 1A, Figure 1B, Figure S1A); these included the three *Ace* resistance substitutions that segregate in the DGRP (I199V [3R_9069054_SNP], G303A [3R_9069408_SNP] and F368Y [3R_9069721_SNP]; Mutero *et al.* 1994; Menozzi *et al.* 2004; Battlay *et al.* 2016) and 2R_8072884_INS, the Accord LTR insertion which differentiates the ancestral *Cyp6g1-M* allele from resistant *Cyp6g1* alleles (Daborn *et al.* 2002; Schmidt *et al.* 2010). Selective sweeps involving *Ace* and *Cyp6g1* have previously been described in the DGRP (Garud *et al.* 2015), and our malathion GWAS identified 128 and 18 associated variants from within boundaries of the *Ace* and *Cyp6g1* sweeps respectively.

To ascertain whether any other selective sweep regions may associate with our phenotypes, we looked for localization of phenotype-associated variants within the most extreme signatures of selection in the DGRP identified by Garud *et al.* (2015) using the 10kb-windowed H12 statistic. Of 273 malathion variants, we observed a total of 151 associated variants within four of the 50 H12 selective sweep peaks. For the 39 permethrin variants, there were no overlaps with any H12 peaks. To assess whether malathion-associated variants were located on the haplotypes driving the selection signals at the four H12 peaks, we assessed their association with the two most common H12 test haplotypes (Figure S2). H1 and H2 (the two most common haplotypes at each sweep peak) are significantly more likely to contain resistant variants for all three *Ace* sites, *Cyp6g1*, and one variant in a third H12 peak (3R:13258656; Fisher’s exact test, *P* < 0.05). However, minor alleles at 3R:13258656 are only observed in H2 and absent from H1, making it unlikely that this variant is the major driver of haplotype structure at this locus.

### Transcriptome associations

To determine the transcriptomic effects of our GWAS candidates, we interrogated datasets generated by Huang *et al.* (2015) for insecticide-associated variants which are also eQTL (genomic variants associated with variation in mean of a particular transcript level) and veQTL (genomic variants associated with variation in variance of a particular transcript level; Tables S3–S6). 53 unique malathion-associated variants (1.25×10^−6^) were eQTL, of which 16 within the *Cyp6g1* sweep boundaries were eQTL of *Cyp6g1* and its tandem paralog *Cyp6g2*. Almost all other malathion-associated eQTL (33), and 42 of the 45 malathion-associated veQTL, mapped to the *Ace* sweep region. Given the extended linkage disequilibrium within the sweep region, these are side-effects of the strong association between malathion phenotypes and *Ace* resistance mutations. Four permethrin-associated variants (2.5×10^−6^) were eQTL and veQTL, three of which were annotated to *Cyp6a23* and were eQTL and veQTL of *Cyp6a23*’s tandem paralog, *Cyp6a17*.

We also tested for associations directly between insecticide phenotypes and Huang *et al.*’s (2015) DGRP transcriptome data. For malathion, 42 transcript associations were identified (*P* < 1.25×10^−4^; Table S7), one of which, *Cyp6g1*, was below the Bonferroni-corrected significance threshold (3.45×10^−7^), and was also the only transcript linked to malathion-associated genomic variants via eQTL. Notably, 10 of the 42 malathion candidate transcripts were among those found by Misra *et al.* (2011) to have their expression altered by twofold or more by ectopic expression of *CncC*. These include *Cyp12d1*, a cytochrome P450 enzyme that confers resistance to DDT and dicyclanil when overexpressed (Daborn *et al.* 2007), and *Jheh1* and *Jheh2*, shown by Guio *et al.* (2014) to increase resistance to malathion when induced. In the case of permethrin, 11 transcript associations were identified (*P* < 2.5×10^−4^; Table S8), none of which were below the Bonferroni-corrected significance threshold (6.89×10^−7^). While *Cyp6a17* transcript levels (of which three permethrin-associated variants are linked by eQTL and veQTL) do not reach significance at 2.5×10^−4^ in any of our transcriptome to phenotype association tests, they have a high rank in all phenotypes (male 3-hour knockdown rank = 26/18137, adjusted r^2^ = 0.060; female 3-hour knockdown rank = 4/18139, adjusted r^2^ = 0.064; male 24-hour mortality rank = 26/18137, adjusted r^2^ = 0.057; female 24-hour mortality rank = 26/18139, adjusted r^2^ = 0.066).

### Structural variation in candidate genes

**Figure 2:**
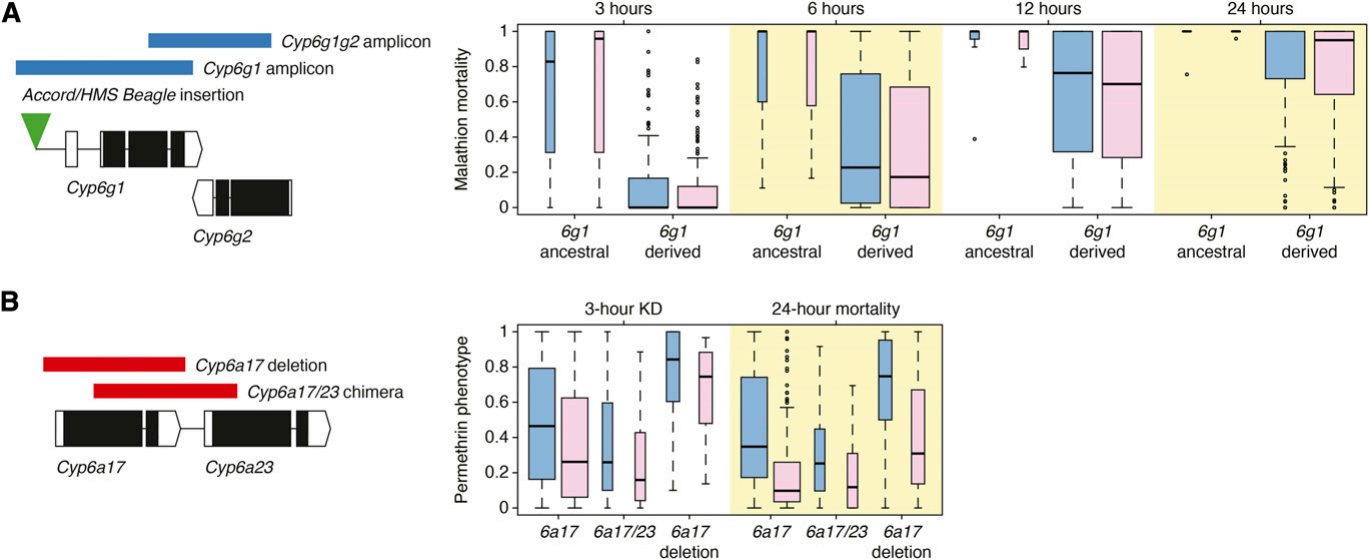
Structural variation in candidate insecticide resistance genes. DGRP structural variation in insecticide resistance candidates (A) *Cyp6g1* and (B) *Cyp6a17* and *Cyp6a23*. Box plots show phenotype distributions among DGRP lines at each phenotype, grouped by structural variant allele. Blue plots represent males and pink plots represent females. Mean phenotypes for derived *Cyp6g1* alleles and *Cyp6a17* deletion alleles are significantly different from reference alleles in all relevant phenotypes (Table S9).

Structural variation among DGRP lines has previously been reported for association study candidates *Cyp6g1*, *Cyp12d1*, *Jheh1/Jheh2* and *Cyp6a17/Cyp6a23* (Zichner *et al.* 2013; Good *et al.* 2014; Guio *et al.* 2014; Figure 2, Figure S3). Of these structural variants, only *Cyp6g1* is directly called in DGRP genotype data (2R_8072884_INS encodes the presence of the *Accord* transposable element insertion, present in all derived *Cyp6g1* alleles). Therefore, the incidences of structural variants were manually tested for association against the relevant insecticide phenotype, and significant associations were found with *Cyp6g1* derived alleles in the case of malathion, and the *Cyp6a17* deletion allele in the case of permethrin (two tailed *t*-test assuming unequal variances, *P* < 0.05; Figure 2).

Discordant paired-end read mapping over the *Cyp6g1* and *Cyp6g2* loci shows that the two gene amplification events described in Schmidt *et al.* (2010) are present in the *Cyp6g1-AA* and *Cyp6g1-BA* alleles among DGRP lines. However, read depth across the region suggests substantial variation in copy number of both these amplicons, implying 1-5 copies of the *Cyp6g1* amplicon, and 1-10 copies of the partial *Cyp6g1g2* amplicon, which are correlated with transcript levels of both *Cyp6g1* and *Cyp6g2* (Figure S4).

### Knockout of Cyp6g1 increases susceptibility to three organophosphate insecticides

**Figure 3:**
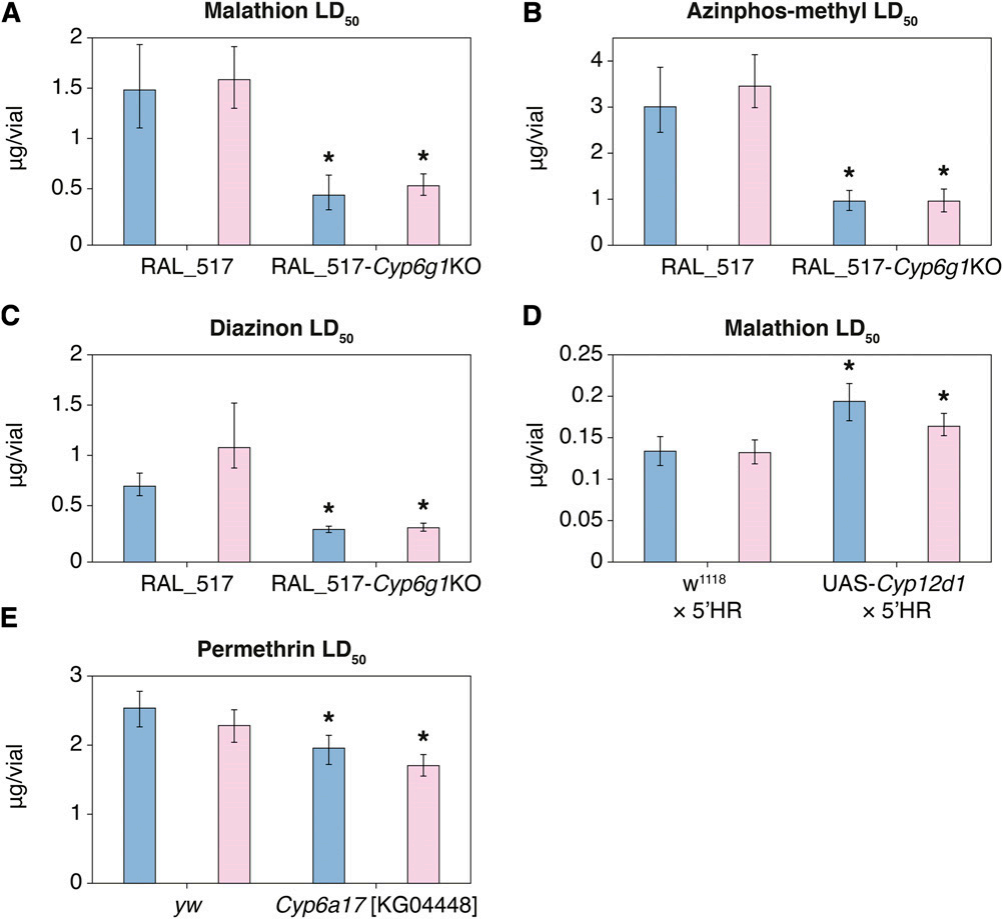
Functional validation of insecticide resistance candidates. Insecticide LD_50_s for both male and female adults. Error bars represent 95% confidence interval. Blue bars represent males and pink bars represent females. CRISPR knockout of *Cyp6g1* in the DGRP line RAL_517 background significantly increases susceptibility to organophosphate insecticides malathion, azinphos-methyl and diazinon. Transgenic overexpression of *Cyp12d1* with the *6g1*HR-GAL4 driver significantly increases resistance to malathion. Gene Disruption Project line *Cyp6a17^KG04448^* shows increased permethrin susceptibility.

Natural variation at *Cyp6g1* contributes to resistance to a range of insecticides including DDT (Schmidt *et al.* 2010), azinphos-methyl (Battlay *et al.* 2016) and imidacloprid (Denecke *et al.* 2017) and also ranks at the top of our genomic and transcriptomic association tests with malathion phenotypes. We verified *Cyp6g1* involvement in malathion resistance using RAL_517-*Cyp6g1*-KO, a DGRP line in which the natural resistance allele *Cyp6g1-BA* is knocked out (Denecke *et al.* 2017). We observed a decrease in malathion median lethal dose (LD_50_) of approximately two thirds in both male and female RAL_517-*Cyp6g1*-KO flies when compared to unmodified RAL_517 flies (Figure 3A).

Battlay *et al.* (2016) previously demonstrated that *Cyp6g1* overexpression, both transgenic (using GAL4-UAS overexpression) and among DGRP lines, was associated with resistance in larvae to the organophosphate azinphos-methyl. Pyke *et al.* (2004) mapped resistance to another organophosphate, diazinon, in an Australian natural population of *D. melanogaster* to a region containing *Cyp6g1*. Here we also present toxicological assays of RAL_517-*Cyp6g1*-KO and RAL_517 that demonstrate that the removal of the natural *Cyp6g1-BA* resistance allele from RAL_517 significantly reduces resistance to azinphos-methyl (Figure 3B) and diazinon (Figure 3C) in male and female adults.

### Cyp12d1 overexpression increases malathion resistance

Increased expression of *Cyp12d1* has previously been linked with resistance to the insecticides DDT and dicyclanil (Pedra *et al.* 2004; Daborn *et al.* 2007; Gellatly *et al.* 2015). In this study, we detected an association between male mortality at 24 hr and transcript level of *Cyp12d1-p* (adjusted r^2^ = 0.12; *P* = 4.80×10^−6^), one of the two copies of *Cyp12d1* present in the *y*; *cn bw sp*; genome. Flies transgenically overexpressing *Cyp12d1* had significantly higher malathion LD_50_s (∼30% and ∼20% increases for males and females respectively) than control crosses (Figure 3D).

### Cyp6a17 disruption increases permethrin susceptibility

Top permethrin-associated genomic variants are eQTL of *Cyp6a17*, a locus at which two deletion variants exist in the DGRP. To test the hypothesis that *Cyp6a17* contributes to permethrin resistance, we obtained a Gene Disruption Project line (Bellen *et al.* 2011), *Cyp6a17^KG04448^*, in which a *P*-element construct had been inserted into the coding region of *Cyp6a17*, early in the first exon. We found significantly reduced permethrin LD_50_s in both males and females from this line when compared to control flies (Figure 3E).

## Discussion

In this study, the DGRP was assayed for resistance to malathion and permethrin, representatives of two of the most widely used insecticide classes, organophosphates and pyrethroids. Sexes were phenotyped separately and scored at multiple time points, increasing the resolution with which variants contributing to resistance could be identified. The majority of associations significant at the genome-wide association threshold are private to a single sex, including variants in and around a major malathion candidate, *Cyp6g1*, which were only significant in females at the three-hour mortality phenotype (Figure S1). Different sex and time point phenotypes may result in different associated candidates through two avenues: biological effects, like sexual dimorphism or a threshold exposure time required to elicit a response, (*i.e.*, gene induction; Willoughby *et al.* 2006), and statistical effects that are consequences of skewed phenotypic distributions, whereby genes with low minor allele frequencies can be associated at more extreme phenotypes. *Cyp6g1* appears to fall into the latter category, as the ancestral allele at this locus is rare (only present in nine DGRP lines; Battlay *et al.* 2016) and our transgenic results demonstrate that *Cyp6g1* confers resistance to malathion and other organophosphates at a range of doses in both sexes (Figure 3A-C).

Evidence suggests that insecticides have played a large role in recent selection in *D. melanogaster*. Here, we find malathion-associated variants in four of the top 50 H12 selective sweep peaks identified in the DGRP by Garud *et al.* (2015). However only associations in the windows containing *Cyp6g1* and *Ace* display distributions in haplotype structure congruent with the selective sweep windows containing them (Figure S2). There is strong evidence to support the hypothesis that both *Cyp6g1* and *Ace* are the targets of selection in multiple populations including the ancestral population of the DGRP (Catania *et al.* 2004; Schmidt *et al.* 2010; Karasov *et al.* 2010; Garud *et al.* 2015; Garud and Petrov 2016), and the link to malathion resistance is now compelling.

Of the four substitutions in *D. melanogaster Ace* known to confer enzymatic insensitivity and hence resistance to organophosphate and carbamate insecticides (Menozzi *et al.* 2004), the three most common are present in DGRP lines (I199V, G303A and F368Y). Each of these three nonsynonymous sites independently achieved Bonferroni-significant associations with male and female malathion mortality at 6 hr. Only three combinations of these *Ace* alleles are present in the DGRP at moderate frequencies: The ancestral, susceptible *Ace* haplotype (*Ace*-IGF), and two resistant *Ace* substitution haplotypes (*Ace*-VGF (one substitution) and *Ace*-VAY (three substitutions). *Ace*-VGF and *Ace*-VAY enzymes have inhibitory constants of 6.4 and 32 to malaoxon (the activated form of malathion) respectively (Menozzi *et al.* 2004), and we find that DGRP population mean malathion mortalities for each haplotype corresponds to these relationships, suggesting that the previously characterized role of *Ace* resistance substitutions explains the strong malathion associations detected within *Ace* and the surrounding haplotype. In contrast, DGRP GWAS of resistance to the organophosphate azinphos-methyl did not detect strong associations with these alleles (Battlay *et al.* 2016). This is likely due to the lower inhibitory constants of these alleles to azinphos-methyl than malathion; *Ace*-VGF actually reduces the inhibitory constant to 0.92, and *Ace*-VAY only increases it to 4.8 (Menozzi *et al.* 2004).

Derived alleles of *Cyp6g1* are associated, through both genomic and transcriptomic variation, with malathion resistance, and the link between malathion-associated genomic variants and *Cyp6g1* expression is demonstrated by eQTL mapped by Huang *et al.* (2015; Table S4). This study adds to the mounting evidence that *Cyp6g1* overexpression in wild populations confers resistance to multiple organophosphate insecticides (Kikkawa 1961; Pyke *et al.* 2004; Battlay *et al.* 2016), and that organophosphate selection may be a more likely explanation than DDT for the sweep observed at the *Cyp6g1* locus (Schmidt *et al.* 2017). Moreover, we find that the DGRP harbors greater allelic diversity at *Cyp6g1* than had previously been described at the locus, and that these additional structural variants, along with those previously characterized, are correlated with differences in transcription of *Cyp6g1* and downstream *Cyp6g2*.

This work also implicates cytochrome P450s in resistance to permethrin. DGRP variants most strongly associated with permethrin map to a region on chromosome 2R containing nine P450 genes, with peaks over *Cyp6a23* and *Cyp317a1*. Three of these variants are annotated by Huang *et al.* (2015) as eQTL and veQTL of *Cyp6a23*’s tandem paralog, *Cyp6a17*, and structural variation in the DGRP has previously been described involving *Cyp6a17* and *Cyp6a23* (Zichner *et al.* 2013; Good *et al.* 2014). Two deletions in this region are present among DGRP lines, one creates a single chimeric gene comprised of *Cyp6a17* and *Cyp6a23* sequence. In the other, *Cyp6a17* is deleted, save for a small section which exists as a gene conversion in the otherwise intact *Cyp6a23*. Due to the homology between these genes, this gene conversion introduces only four nucleotide changes and a single non-synonymous substitution in *Cyp6a23* (Good *et al.* 2014). Among DGRP lines, it is this deletion of *Cyp6a17* that is associated with increased susceptibility to permethrin (Figure 2B). This is congruent with the finding that when *Cyp6a17* is disrupted in *Cyp6a17^KG04448^*, it leads to relative susceptibility (Figure 3E). It is worth noting that the susceptible allele appears to be the derived state, as the duplication leading to the original divergence *Cyp6a17* and *Cyp6a23* seems to be at least as old as the divergence between *D. melanogaster* and *D. ananassae* (Good *et al.* 2014).

The transcription level of another P450, *Cyp12d1-p*, was associated with male 24-hour malathion mortality, and transgenic overexpression of the gene confers malathion resistance in both male and female adults (Figure 3D). Interestingly, we do not observe a strong association at either the genomic level (Table S9) or the transcriptome level (Table S6) with *Cyp12d1-d*, present as a duplicated paralog in 24% of DGRP lines. Coding (three amino acids differentiate *Cyp12d1-p* and *Cyp12d1-d* in the reference genome) or expression pattern differences between the genes may explain this observation. Alternatively, *Cyp12d1-p*’s significance may be inflated by its strong correlation with a group of co-regulated genes that are induced by oxidative stress. We found that ten of the top malathion-associated transcripts are among those known to be regulated by CncC (Misra *et al.* 2011).

Two more CncC-activated transcripts associated with malathion phenotypes are *Jheh1* and *Jheh2*. Guio *et al.* (2014) demonstrated that the insertion of *Bari1* upstream of *Jheh1* and *Jheh2* increases the inducibility of these genes in response to oxidative stress, which results in an increased resistance to malathion. In this study, we found associations between constitutive transcript levels of *Jheh1* and *Jheh2* and male malathion mortality at 24 hr (adjusted r^2^ = 0.10; *P* = 4.52×10^−5^) and 3 hr (adjusted r^2^ = 0.10; *P* = 4.62×10^−5^) respectively. However, we did not find that the presence of the *Bari-Jheh* insertion was significantly associated with malathion mortality at any of our four time points, for either sex (Figure S3A; Table S9). This is plausibly due to differences in the exposure times between our study (up to 24 hr) and the assays used in Guio *et al.* (2014; up to 214 hr). It makes sense that in our more acute assays, the baseline expression level of expression of *Jheh1* and *Jheh2* would be important, whereas over longer assay periods induction capacity would play a more important role.

An important question in DGRP insecticide resistance studies is the applicability of findings in this subset of variation to populations worldwide. *Ace* and *Cyp6g1* resistance alleles have been identified in a range of populations, as have the footprints of their selection (Catania *et al.* 2004; Karasov, Messer and Petrov 2010; Schmidt *et al.* 2010; Garud and Petrov 2016). This is further reinforced by interrogation of the Drosophila Genome Nexus (DGN; Lack *et al.* 2016), which reveals *Ace* and *Cyp6g1* allele frequencies comparable to the DGRP in many populations around the world (Table S10). The structural variants at *Jheh1/2/3*, *Cyp12d1* and *Cyp6a17/23* have also been described in populations outside the DGRP (González, Macpherson and Petrov 2009; Kolaczkowski *et al.* 2011; Chakraborty *et al.* 2018).

In this investigation of insecticide resistance in the DGRP using an organophosphate and a pyrethroid insecticide we saw stark differences in the genes involved as well as the evidence for a selective response to the compounds. The top candidates from malathion GWAS correlate with peaks of selection in the DGRP population, making organophosphate resistance a highly credible selective pressure on the population ancestral to the DGRP. Conversely, *Cyp6a17*, our top permethrin candidate, does not lie within a H12 selective sweep peak, nor would we expect the allele described to be the target of positive insecticide-based selection, given it increases susceptibility to permethrin. However, *Cyp6a17* ranks fourth among *D. melanogaster* P450s (after *Cyp6a13*, *Cyp6a2*, and *Cyp6a14*) in similarity to malaria vector *Anopheles funestus CYP6P9a* and *CYP6P9b*. Naturally occurring duplications of each of these genes are associated with pyrethroid resistance in *A. funestus* (Wondji *et al.* 2009), and selective sweeps have been described at *CYP6P9a* in response to pyrethroid-based malaria interventions (Barnes *et al.* 2017).

An emerging picture of insecticide resistance, informed by results from DGRP studies as well as investigations in pest insect species (Joußen *et al.* 2012; Faucon *et al.* 2015) is that complex structural variation and high allelic diversity, along with selective sweep signatures, are common in genes contributing to resistance.
